# Mental health professionals’ family-focused practice with families with dependent children: a survey study

**DOI:** 10.1186/s12913-017-2761-7

**Published:** 2017-12-08

**Authors:** Patraporn Tungpunkom, Darryl Maybery, Andrea Reupert, Nick Kowalenko, Kim Foster

**Affiliations:** 1 Thailand Centre for Evidence Based Health Care: a JBI Centre of Excellence, and Mental Health Care Centre, Faculty of Nursing, Chiang Mai University, Chiang Mai, 50200 Thailand; 2 Monash University Department of Rural Health, and Faculty of Medicine, Nursing and Health Sciences, Monash University, PO Box 973, Moe, VIC 3825 Australia; 3 Krongold Clinic, Faculty of Education, Monash University, Clayton, VIC 3168 Australia; 4 CAMHS and University of Sydney, Level 2, CHC, 2a Herbert St, St Leonard’s, NSW 2065 Australia; 5 Australian Catholic University & NorthWestern Mental Health, Royal Melbourne Hospital, Grattan St., Parkville, VIC 3050 Australia

**Keywords:** Children of parents with mental illness, Family-focused practice, Parenting, Mental health professionals, Survey

## Abstract

**Background:**

Many people with a mental illness are parents caring for dependent children. These children are at greater risk of developing their own mental health concerns compared to other children. Mental health services are opportune places for healthcare professionals to identify clients’ parenting status and address the needs of their children. There is a knowledge gap regarding Thai mental health professionals’ family-focused knowledge and practices when working with parents with mental illness and their children and families.

**Methods:**

This cross –sectional survey study examined the attitudes, knowledge and practices of a sample (*n* = 349) of the Thai mental health professional workforce (nurses, social workers, psychologists, psychiatrists) using a translated version of the Family-Focused Mental Health Practice Questionnaire (FFMHPQ).

**Results:**

The majority of clinicians reported no training in family (76.8%) or child-focused practice (79.7%). Compared to other professional groups, psychiatric nurses reported lower scores on almost all aspects of family-focused practice except supporting clients in their parenting role within the context of their mental illness. Social workers scored highest overall including having more workplace support for family-focused practice as well as a higher awareness of family-focused policy and procedures than psychiatrists; social workers also scored higher than psychologists on providing support to families and parents. All mental health care professional groups reported a need for training and inter-professional practice when working with families.

**Conclusions:**

The findings indicate an important opportunity for the prevention of intergenerational mental illness in whose parents have mental illness by strengthening the professional development of nurses and other health professionals in child and family-focused knowledge and practice.

## Background

Mental illness is one of the most significant global health concerns. The burden of mental disorders continues to grow with substantial social, human rights and economic consequences [1]. Worldwide, an estimated 300 million people are affected by depression, 21 million by schizophrenia, and 60 million by bipolar disorder [1], accounting for 7.4% of all disability-adjusted life years worldwide [2] and 16.2% of the burden of disease in Thailand [3]. Mental illness affects not only the individual. In Thailand, almost all people with mental illness live with their family, who provide the main support [4, 5]. Family members, especially children, are inevitably impacted. This study investigated how mental health professionals in Thailand support mental health clients who are parents and their children.

Prevalence estimates indicate that between 20 and 30% of people with mental illness have children [6], and 21–23% of children live with a parent with a mental illness [7]. A meta-analysis found that children whose parents have serious mental illness are at significant risk (up to 55%) of developing a mental illness themselves [8]. Likewise, Hosman, van Doesum and van Santvoort [9] estimate that the risk of developing mental disorders among these children ranges from 41% to 77%, while Maybery, Reupert, Goodyear, Ritchie and Brann [10] found that these children are at two to five times greater risk of developing a mental health problem (depending on parental diagnosis). Outcomes for children depend on a range of interconnecting risk/protective factors including genetic vulnerability as well as child, parent, family and social environment factors [7–11]. However, many of these factors are responsive to intervention. Indeed, a meta-analysis found that interventions in this area reduce children’s risk of acquiring a mental illness by up to 40% [12].

Family members do not, however, necessarily receive education and support from healthcare professionals. A study in German, Austrian and Swiss psychiatric institutions found that only 2% of family members received any form of psycho-education [13]. Several international studies have examined the ways in which different healthcare professions interact with these parents and their children. In Australia for example, Maybery and Reupert [14] found that while various mental health professionals considered it part of their role to work with clients’ children, they reported significant time and resource limitations as well as skill and knowledge deficits. A Finnish study found that most of the 310 nurses surveyed did not meet children of their clients regularly, although they reported discussing clients’ children with them and collected information about the children of their clients [15]. In Greece, Bibou-Nakou [16] found that adult mental health workers did not know enough about the developmental needs of children and so were unable to talk to their clients about their children. In the US, it has been suggested that mental health workers either focus on the child (e.g. in terms of child protection) or the adult (e.g. for his or her mental health needs or parenting capacity [17]. In Canada, while there have been several policy documents somewhat related to this area (e.g. Rising to the Challenge: A Strategic Plan for the Mental Health and Well-being for Manitobans, 2011), children who live with a parent with a mental illness are not typically specified [17]. Another study compared 343 Irish and 155 Australian psychiatric nurses and found that while both samples were not particularly family-focused, the Australian cohort was generally more confident and reported more skills and knowledge when working with clients on their parenting responsibilities and with their client’s children [18]. This is in part likely due to mental health policy and service differences between the two countries [18].

Other studies have examined specific professional differences in family-focused practice. One seminal study’s findings were that social workers and psychiatrists spend more time with families than psychologists who, while they supported the principle of family-focused work, actually spent little time with families as part of treatment [19]. In a more recent study, social workers engaged with parents and children more than psychologists and both groups more than psychiatric nurses, “…who performed consistently the lowest on direct family care, compared to both social workers and psychologists” ([20], p. 608). Profession type has also been highlighted in respect to professional development requirements. Delphi study findings indicated that psychologists need to know more about parenting, general practitioners more about supporting families, and social workers and nurses need to know more about child development [21]. Predominately, previous work has focused on psychiatric nurses. There has been no prior research investigating the family-focused practices of psychiatrists in reference to clients who are parents and their children. This gap in knowledge will be addressed in the current study.

A range of barriers for family-focused practice (FFP) are reported in the literature, especially in regard to worker skill, knowledge and confidence [22]. On this basis, the authors argued that training for mental health professionals is critical when building a family-focused workforce. One preliminary study demonstrated short term improvements in clinicians’ knowledge, skill and confidence following training in a family-focused training package [23]. This evaluation, however, involved specific and targeted training, comprised a small sample (*n* = 27), and did not include longer term follow-up of the impact of the training. The current study explores how Thai mental health professionals, including psychiatrists, work with clients on parenting issues and with clients’ children. This is the first such study in Thailand and the first to include psychiatrists. Family-focused practice involves support directed to the whole family unit, including the person with mental illness and their children [24]. Specific practices include the identification of family members (especially children), the assessment of family functioning, the delivery of psychoeducation to family members, the provision of practical, emotional and social support to the family and liaising between services on behalf of the family [24]. Identifying family-focused practice in Thailand has the potential to inform mental health policy and professional development programs as well as establishing performance indicators for different workforce groups in this area. The current study aimed to investigate the attitudes, knowledge and practices in a sample of the Thai mental health professional workforce, and to provide initial benchmarking data regarding family-focused practice in this workforce. The study also aimed to identify possible differences between the professional groups in Thailand and the relationship of previous family and child-focused training and family-focused practice to family-focused practices.

## Methods

A cross-sectional survey design was used to determine clinicians’ attitudes, knowledge and practices in family-focused practice using a translated version of the Family-Focused Mental Health Practice Questionnaire (FFMHPQ).

### Instrument and translation

The 53 item Family-focused Mental Health Practice Questionnaire (FFMHPQ) [25] was used to investigate the attitudes, knowledge, and family-focused practices of participants. The FFMHPQ has excellent face and content validity and Cronbach’s alpha coefficients are between 0.70–0.90 for most subscales. The FFMHPQ has been used previously to benchmark family-focused practice in different services, to audit practice in adult mental health settings [26] and to evaluate training [27]. For the current study, the FFMHPQ was translated from English to Thai and back-translated into English following the rigorous cross-cultural translation procedure recommended by Beaton & Guillemin [28]. Two translators with Thai as first language independently translated the questionnaire from English to Thai and reached consensus on a combined version. Two translators with English as first language then back-translated the combined version. The project team and translators reached consensus on a final version of the translated FFMHPQ. Prior to the larger data collection, an initial pilot of the translated version was carried out with twenty mental health professionals across the study sites to ensure that the translated version was appropriate and that its items, design and layout were relevant in the Thai context [28]. Participants voluntarily completed the questionnaire and were interviewed by a Thai research assistant about the design and layout of the questionnaire, how easy/difficult it was to answer the questions, and what they thought was meant by the questions and chosen responses [28]. Minor changes to layout and wording were made as a result.

### Sample and setting

In Thailand, when acutely mentally unwell, individuals are admitted to hospital for a limited stay and then discharged home to rehabilitate. Buddhist beliefs are an important cultural influence, including an understanding of caregiving in the context of Karma, boon (merit) and babzb (demerit), and dharma (Buddhist teaching). These beliefs underpin family members’ maintenance of compassion and acceptance in caregiving for their relatives [29]. While the mental health care of these children and families is not specifically targeted, promotion of mental health and prevention of mental illness across the life span is part of the country’s Mental Health Policy and Service Plan [30]. In Thailand, as in other countries, there is significant subjective and objective caregiving burden for family members caring for their relative with a mental illness [29]. Hence, family members including children need substantial information and family-focused support.

The proportion of mental health professionals per 100,000 population in Thailand is psychiatrists 2.05 (*n* = 102); child and adolescent psychiatrists 0.78 (*n* = 35); psychiatric nurses 26.76 (*n* = 1350), divided into Bachelor degree with psychiatric mental health nursing certification 14.13 (*n* = 730), child and adolescent psychiatric nurse 4.13 (*n* = 192), and Masters degree in psychiatric and mental health nursing 8.46 (*n* = 428); psychologists 4.64 (*n* = 241), clinical psychologists 2.02 (n = 102), [31] and social workers 0.13 (*n* = 85) [32]. Psychiatric nurses are the largest group in the workforce. In Thailand, psychiatric nurse roles are divided into two; one is to take care of clients in hospital and provide comprehensive assessment, nursing care, coordinate with other professionals, assess for medication side-effects, provide individual and/or group activity therapy, and prepare clients and family in skills needed when the client is discharged. The other role is community–focused, and includes coordinating with other organizations in the community and conducting community home visits. Psychiatric nurses with Masters degrees are qualified to provide additional individual and/or group psychotherapy. The role of psychiatrists is to diagnose and treat psychiatric disorders. Psychologists provide psychological assessment to help psychiatrists to clarify psychiatric diagnosis and provide individual and/or group psychotherapy. Social workers assess social problems related to the mental illness, provide counselling to clients and family/caregivers and family, including their right to treatment and the cost of treatment and financial support when needed. They also assess social and community aspects related to client relapse [33].

A power calculation based on average standard deviation in an Australian sample with 90% power to detect a difference in groups with a level of significance of 0.05 for a two sample test on mean scores, determined that a sample size of *n* = 300 was sufficient to achieve the aims of the study and provide adequate power to determine FFMHPQ scores [25]. The FFMHPQ was administered to a total convenience sample of 402 mental health professionals from two large psychiatric hospitals in the Northern and North Eastern regions of Thailand. These settings provide inpatient and outpatient department services primarily for adult clients.

In total, 349 staff completed the survey (a response rate of 87%). The majority were female (*n* = 296/84.5%) and the average age was 41.99 years (23–60 years). The majority had Bachelor degrees (*n* = 173/49.6%). Most were psychiatric nurses (*n* = 295/84.5%) working full time (*n* = 339/97.1%), predominantly in a clinical role (*n* = 310/88.8%) in inpatient settings (*n* = 207; 59.5%). The average length of time working in their current role was 12. 69 years (1 month - 38 years), and working in mental health was 18.67 years (2 months - 49 years). Participants had worked as an adult clinician for an average of 16.97 years (1 month - 38 years); 3.20 years working with child/children (0–37 years); and 4.97 years working with family (0–38 years).

### Ethical procedure and consent

Ethics approval was gained from the Thailand Research Council and the relevant Universities and Hospitals prior to study commencement. Hospital unit managers gave permission for the Thai research assistants to administer the questionnaire in each psychiatric unit at each site. Research assistants provided information sessions for staff, and written study information. Staff anonymously completed and returned paper versions of the questionnaire. Consistent with accepted research practice, completion of the questionnaire was taken as implied consent.

### Analysis

Means and standard deviations (SD) were calculated for each of the subscales for all participants, and ANOVA computations undertaken to examine the differences between professions. Post-hoc tests were also run in order to determine any profession differences in scores. A series of between subjects ANOVAs were then undertaken to compare those participants who had had previous family or child-focused training with those who had not had such training.

## Results

Scores for ANOVA computations for each sub-scale in relation to the four professions are shown in Table 1.

**Table 1:** Means and Standard Deviation of Subscales by Professional Background

Subscales	Professional Status				Total scores	Post Hoc Tests
	Psychiatric Nurse (n = 295)	Psychologist (*n* = 17)	Social Worker (*n* = 14)	Psychiatrist (*n* = 12)		
Workplace support for family-focused practice	3.88 (1.91)*	3.88 (2.24)*	5.61 (1.19)*	3.21 (1.95)*	3.93 (1.93)	1 < 3, 2 < 3, 3 > 4
Location issues in relation to family-focused practice	3.68 (1.79)*	4.65 (2.14)*	5.25 (1.15)*	3.00 (1.94)*	3.77 (1.83)	1 < 2, 1 < 3, 3 > 4, 4 < 2
Time and workload	3.74 (1.70)*	5.08 (1.54)*	6.07 (0.87)*	3.42 (1.66)*	3.89 (1.74)	1 < 2, 1 < 3, 3 > 4, 4 < 2
Family-focused policy and procedure	3.95 (1.47)*	3.50 (1.47)*	4.79 (0.89)*	2.92 (1.06)*	3.93 (1.46)	1 < 3, 1 > 4, 3 > 2, 3 > 4
Family-focused professional development opportunities	4.28 (1.71)*	5.03 (1.64)	5.43 (1.26)*	4.42 (1.29)	4.73 (1.70)	1 < 3
Coworker Support	3.88 (1.67)*	4.94 (1.21)*	5.43 (1.42)*	4.63 (1.52)	4.03 (1.67)	1 < 2, 1 < 3
Providing support to the family & client for parenting	4.25 (1.41)	3.87 (1.50)*	4.92 (1.02)*	4.57 (0.62)	4.27 (1.39)	3 > 2
Worker confidence in family-focused practice	4.29 (1.66)*	5.29 (1.36)*	5.94 (0.99)*	5.56 (1.11)*	4.46 (1.66)	1 < 2, 1 < 3, 1 < 4
Providing support to family & children	4.60 (1.81)*	4.43 (2.01)*	6.30 (0.60)*	5.11 (1.01)	4.68 (1.79)	1 < 3, 3 > 2
Engaging with family	3.63 (1.48)*	4.20 (1.17)	5.12 (1.18)*	4.17 (0.78)	3.74 (1.47)	1 < 3
Assessing impact of parental mental illness on the child	3.72 (1.83)*	4.79 (1.96)*	4.71 (1.34)*	4.96 (1.15)*	3.86 (1.83)	1 < 2, 1 < 3, 1 < 4
The need for further training	5.28 (1.52)	5.76 (1.29)	5.88 (0.93)	4.85 (1.44)	5.32 (1.50)	–
Skill and knowledge	4.02 (1.46)*	4.76 (1.51)*	5.30 (0.94)*	5.37 (1.38)*	4.16 (1.48)	1 < 2, 1 < 3, 1 < 4
Service availability	3.61 (1.73)*	5.00 (1.44)*	4.75 (1.47)*	4.54 (1.57)	3.76 (1.74)	1 < 2, 1 < 3
Promoting family connectedness	4.05 (1.67)*	4.59 (1.73)	5.07 (1.42)*	4.75 (1.67)	4.14 (1.67)	1 < 3
Referrals for family members	2.72 (2.01)*	2.74 (2.28)*	4.71 (1.42)*	3.17 (2.27)	2.82 (2.05)	1 < 3, 3 > 2
Inter-professional practice	5.52 (1.32)	5.90 (1.07)	6.11 (0.75)	6.08 (0.79)	5.58 (1.28)	–
Supporting parenting within context of mental illness	3.14 (1.39)*	2.72 (1.56)	2.37 (0.87)*	1.96 (1.04)*	3.05 (1.39)	1 > 3, 1 > 4

*P < 0.05

With some exceptions, social workers scored higher than other professions on many variables (e.g. family-focused workplace support, policy and procedures). Social workers scored higher than nurses on almost all items except supporting parenting within the context of the person’s mental illness. Social workers also scored higher than psychiatrists on variables such as obtaining appropriate workplace support and adequate time workload for family work, as well as family-focused policy and procedures, and higher than psychologists on providing support to families and parents. Interestingly, psychiatric nurses scored higher on supporting parenting within the context of mental illness than other professions, especially social workers and psychiatrists.

Conversely, nurses scored lowest on most family-focused practices, including worker confidence, assessing the impact on the child, and family-focused skill and knowledge. There were several other variables such as time and workload, workplace support and location issues where they scored low but equivalent to psychiatrists. Perhaps the most important finding was that they scored lower than all other professions on worker confidence, assessing the impact of a parent’s mental illness on the child, and family-focused skill and knowledge. A notable exception was that nurses scored higher than psychiatrists in terms of policy and procedures and supporting parenting within the context of the person’s mental illness. Further to this, psychiatrists scored lowest on policy and procedures but highest of any profession on skill and knowledge. Psychologists scored between the nurses and social workers on many variables. All professions highlighted a need for training and inter-professional practice.

Table 2 shows descriptive statistics and significant differences (in bold) for those who had/not received previous family or child-focused training.

**Table 2:** Means and Standard Deviation in Family-Focused and Child-Focused Practice Training

Subscales	Training in Family-Focused Practice		Training in Child-Focused Practice	
	Yes (*n* = 74)	No (*n* = 268)	Yes (*n* = 62)	No (*n* = 278)
Workplace support for family-focused practice	4.38 (2.04)*	3.81 (1.88)	4.54 (1.75)*	3.80 (1.95)
Location issues in relation to family-focused practice	4.22 (1.84)*	3.69 (1.80)	4.29 (1.69)*	3.71 (1.84)
Time and workload	4.49 (1.81)*	3.78 (1.69)	4.62 (1.59)*	3.77 (1.73)
Family-focused policy and procedure	4.22 (1.30)	3.85 (1.50)	4.02 (1.38)	3.91 (1.49)
Family-focused professional development opportunities	4.78 (1.54)*	4.27 (1.70)	4.91 (1.53)*	4.25 (1.69)
Coworker Support	4.68 (1.68)*	3.88 (1.61)	4.81 (1.46)*	3.88 (1.65)
Providing support to the family and client for parenting	4.55 (1.23)	4.21 (1.43)	4.52 (1.17)	4.24 (1.44)
Worker confidence	5.25 (1.44)*	4.27 (1.64)	5.46 (1.23)*	4.26 (1.66)
Support to carers and children	5.23 (1.54)*	4.50 (1.85)	5.12 (1.54)*	4.56 (1.85)
Engagement issues	4.05 (1.45)*	3.66 (1.44)	4.15 (1.24)*	3.56 (1.48)
Assessing the impact of parental mental illness on the child	4.37 (1.86)*	3.76 (1.80)	4.77 (1.70)*	3.68 (1.80)
The need for further training	5.44 (1.39)	5.32 (1.50)	5.68 (1.02)*	5.26 (1.55)
Skill and knowledge	5.05 (1.23)*	3.93 (1.45)	5.08 (1.14)*	3.97 (1.47)
Service availability	4.20 (1.62)*	3.67 (1.76)	4.31 (1.77)*	3.66 (1.72)
Promoting family connectedness	4.87 (1.49)*	3.97 (1.65)	4.81 (1.37)*	4.02 (1.69)
Referrals for family members	3.26 (2.16)	2.74 (2.00)	3.61 (1.95)*	2.69 (2.03)
Inter-profession practice	5.78 (1.06)	5.55 (1.33)	6.01 (0.67)*	5.51 (1.37)
Supporting parenting with the context of mental illness	2.68 (1.20)*	3.15 (1.41)	2.63 (1.19)*	3.13 (1.40)

*P < 0.05

All participants who had received previous training in family and child-focused practice scored higher on many of the FFMHPQ items than those who did not. This applied to both family and child-focused training. There was no difference in the need for training in family-focused practice, making referrals for family members, and inter-professional practice between participants with previous training and those without.

## Discussion

This is the first study to investigate family-focused practices of mental health professionals in Thailand, and the first study reported internationally to include psychiatrists. There were substantial differences between professions in terms of family-focused practices (FFP). Social workers engaged in almost all types of FFP consistently more often than psychiatric nurses. Social workers provided more workplace support, and were more aware of family-focused policy and procedures than psychiatrists, and scored higher than psychologists on practices such as providing family and parenting support. This may be because of the scope of practice of social workers in client care teams in Thailand, as they need to liaise with the client, their family, as well as other organizations after the client is discharged home [33].This finding is generally consistent with prior literature which has found that social workers engage in more FFP than psychiatric nurses [20], and most likely reflects social workers’ philosophical orientation to working holistically as well as their undergraduate education [34]. As prior research has not included psychiatry, the current study adds to the knowledge base in this field by indicating that social workers also undertake more FFP compared to psychiatrists.

The largest group of professionals, nurses, scored lowest overall, and specifically on items related to worker confidence, assessing the impact of parental mental illness on children, and family-focused skill and knowledge. Nurses have consistently scored lower in FFP than other health professionals [20] and this likely reflects the relatively little emphasis on family work and training in their undergraduate preparation. There were several other factors such as time and workload, workplace support and location issues where they scored low but equivalent to psychiatrists. These items pertain to not having enough time to work with families, having too high a workload to work with families, not receiving sufficient workplace support, and working in a location that made it difficult to work with families. Interestingly, nurses scored higher on supporting parenting within the context of mental illness than other professions, and this was statistically significant compared to social workers and psychiatrists. This could be because the degree of Bachelor of Nursing Science in Thailand includes Midwifery within the curriculum, which includes knowledge and skills in parenting [35], and is not taught separately as occurs in many other countries.

The most important finding was that nurses scored lower than all other health professionals on the core child and family-focused variables of worker confidence, assessing the impact of a parent’s mental illness on the child, and family-focused skill and knowledge. This might be because the two hospital settings did not provide specific child and adolescent services and therefore this was out of their scope of practice. Similar findings have been reported by nurses in countries such as Ireland [36]. These variables have been identified as important enablers and predictors for family-focused practice [37, 38]. This finding has implications for future workforce development in Thailand, where nurses may require more in-depth training in these particular areas. Psychologists scored between the nurses and social workers on many of the variables, and this is also generally consistent with other studies [17, 20].

Psychiatrists scored lowest on an awareness of family-focused policy and procedures but highest of any profession on family-focused skill and knowledge. There are no prior studies to compare these findings to. Future research should address this gap. There is a shortage of psychiatrists in Thailand, especially child and adolescent psychiatrists who are employed by the Thailand Mental Health Department. Given their identified skill and knowledge, better targeting their professional role might facilitate psychiatrists engaging more in family-focused practice [39]. There are, however, comparatively more psychiatric nurses, psychologists and social workers in the workforce [31, 32]. Consequently, nurses are the profession that mental health clients and their families predominately work with and obtain support from. Considering the large number of nurses in the Thai mental health workforce, their low level of family-focused practice (as highlighted in this study), has important implications for nursing professional development and service delivery to families. Although all the professions expressed the need for training and inter-professional practice, there is a priority need to develop the skills and knowledge for Thai psychiatric nurses to build their confidence to work on family-focused practice, particularly in how to support parents with mental health issues, along with skills in engaging families including children. Although there is a postgraduate child and adolescent psychiatric nursing certificate program in Thailand which develops knowledge and practices in working with children and adolescents with mental illness and their family, it does not focus on children who have parents with mental illness [40]. Further, there are only 192 psychiatric nurses or 4.13 per 100,000 population who are specifically trained in child and adolescent psychiatric nursing. There is an urgent need to implement mental health professional workforce training on working with families where parents have mental illness, and to include curricula on children and families where parents have mental illness within existing postgraduate programs for psychiatric nurses and other mental health professional programs in Thailand.

A further key finding from this research was that all participants who had previously undergone family and/or child focused training scored higher on FFP than those who had not been exposed to such training. This is an under-researched area in the literature and the current finding builds on, and is consistent with, previous research [e.g. 20]. However, it must be noted that these staff most likely self-selected to do the training, and training may in itself not be sufficient to promote workforce capacity in this area. As argued previously by Reupert et al. [38], the necessary policy and organisational procedures (such as identification of clients’ parenting status) need to be in place before training gains can be translated into everyday practices.

### Study limitations

The study is limited to a sample drawn from two psychiatric hospitals in Thailand. Further research is needed with a broader representative population of mental health professionals in Thailand. The views of stakeholders, in particular Thai parents with a mental illness and their children, are lacking and these need to be elicited in future studies to ascertain how their families and parenting responsibilities might be best supported by mental health professionals.

## Conclusions

There is an urgent need to educate Thai mental health workers in family-focused practice. It is recommended that a national survey on the training needs in this area is conducted to inform policy and practice development. The policy of the Thailand Mental Health Department is focused on enhancing mental health promotion and mental illness prevention across the life span [30]. The findings from this study indicate an important opportunity for prevention of intergenerational mental illness in children whose parents have mental illness by strengthening the professional development of nurses and other health professionals in child and family-focused knowledge and practice. Further studies are needed to consider whether prior training in family, parenting, and/or experience working with children is associated with higher levels of family-focused practice. Although prevention is a policy priority for Thailand, the needs of children of parents with mental illness have not yet been specifically identified in mental health policy. Given their substantially increased risk for mental health problems (e.g. Rasic et al., [8]) it is recommended that the needs of children of parents with mental illness, as well as that of their parents and families, are a specific priority area for future healthcare policy and for workforce development.

## Abbreviations

**FFMHPQ**: Family Focused Mental Health Practice Questionnaire
**FFP**: Family Focused Practice
